# Usefulness of superb microvascular imaging for differential diagnosis of malignant cardiac tumours: a case series

**DOI:** 10.1093/ehjcr/ytag343

**Published:** 2026-05-11

**Authors:** Ayaka Mizumoto, Masashi Amano, Yutaka Demura, Takahiro Sakamoto, Yoshiyuki Sumita, Chisato Izumi

**Affiliations:** Department of Clinical Laboratory, National Cerebral and Cardiovascular Center, 6-1 Kishibe-shinmachi, Suita, Osaka 564-8565, Japan; Department of Heart Failure and Transplantation, National Cerebral and Cardiovascular Center, 6-1 kishibe-chinmachi, Suita, Osaka 564-8565, Japan; Department of Clinical Laboratory, National Cerebral and Cardiovascular Center, 6-1 Kishibe-shinmachi, Suita, Osaka 564-8565, Japan; Department of Clinical Laboratory, National Hospital Organization, Osaka National Hospital, 2-1-14 Hoenzaka, Chuo-ku, Osaka 540-0006, Japan; Department of Heart Failure and Transplantation, National Cerebral and Cardiovascular Center, 6-1 kishibe-chinmachi, Suita, Osaka 564-8565, Japan; Division of Cardiology, Faculty of Medicine, Shimane University, 89-1, Enya-cho, Izumo, Shimane 693-8501, Japan; Department of Clinical Laboratory, National Cerebral and Cardiovascular Center, 6-1 Kishibe-shinmachi, Suita, Osaka 564-8565, Japan; Department of Heart Failure and Transplantation, National Cerebral and Cardiovascular Center, 6-1 kishibe-chinmachi, Suita, Osaka 564-8565, Japan

**Keywords:** Superb microvascular imaging, Intratumoural blood flow, Cardiac tumour, Sarcoma, Malignant lymphoma, Case report

## Abstract

**Background:**

Superb microvascular imaging (SMI) can visualize low-velocity blood flow vividly without contrast agents. Superb microvascular imaging could be used to evaluate blood flow in the myocardium or cardiac tumours, which have never been assessed using a conventional echocardiographic probe. Evaluating intratumoural blood flow using SMI may provide supportive haemodynamic characterization for the differential diagnosis of cardiac tumours.

**Case summary:**

This report describes three cases in which SMI was useful for detecting intratumoural blood flow signals and differential diagnosis of cardiac tumours. Intratumoural blood flow signals were detected in Case 1 (pulmonary artery intimal sarcoma) but not in Case 2 (malignant lymphoma). The patient described in Case 3 had a large intracardiac tumour and was too old and frail to undergo surgery; therefore, based on the SMI findings of abundant intratumoural blood flow signals, cardiac sarcoma was presumed, and best supportive care was selected without performing invasive tests or treatment.

**Discussion:**

Rapid differential diagnosis between cardiac sarcoma and malignant lymphoma is important for determining the treatment strategy. Cardiac sarcoma has a poor prognosis and progresses rapidly, whereas cardiac malignant lymphoma can potentially be cured by chemotherapy. Intratumoural blood flow is abundant in patients with cardiac sarcoma but poor in those with malignant lymphoma. Superb microvascular imaging can assist whether blood flow is abundant or poor in a cardiac tumour. Evaluation of intratumoural blood flow using SMI provides supportive haemodynamic characterization for the differential diagnosis of cardiac tumours and may shorten the time to treatment.

Learning points

- Superb microvascular imaging (SMI) can evaluate abundant (cardiac sarcoma) or poor (malignant lymphoma) blood flow in a cardiac tumour.
- Evaluation of intratumoural blood flow using SMI provides supportive characterization for the differential diagnosis of cardiac tumours and may shorten the time to treatment.

## Introduction

Superb microvascular imaging (SMI), which is developed by Canon Medical Systems, is a new imaging technique that can visualize low-velocity blood flow vividly without contrast agents. While both low-velocity blood flow and tissue motion artefacts are removed by conventional filters,^1^ SMI, which is based on an original algorithm, can effectively remove only tissue motion artefacts from the background, thereby preserving the visibility of low-velocity blood flow (see Supplementary material online, *Figure S1*).^2^ We have previously reported cases in which SMI was useful for the evaluation of abnormal structures in the heart.^3^ Superb microvascular imaging could be used to evaluate blood flow in the myocardium or in a cardiac tumour, which has never been assessed using a conventional echocardiographic probe. This technique may provide supportive haemodynamic characterization for the differential diagnosis of cardiac tumours.

## Summary figure

**Figure:**
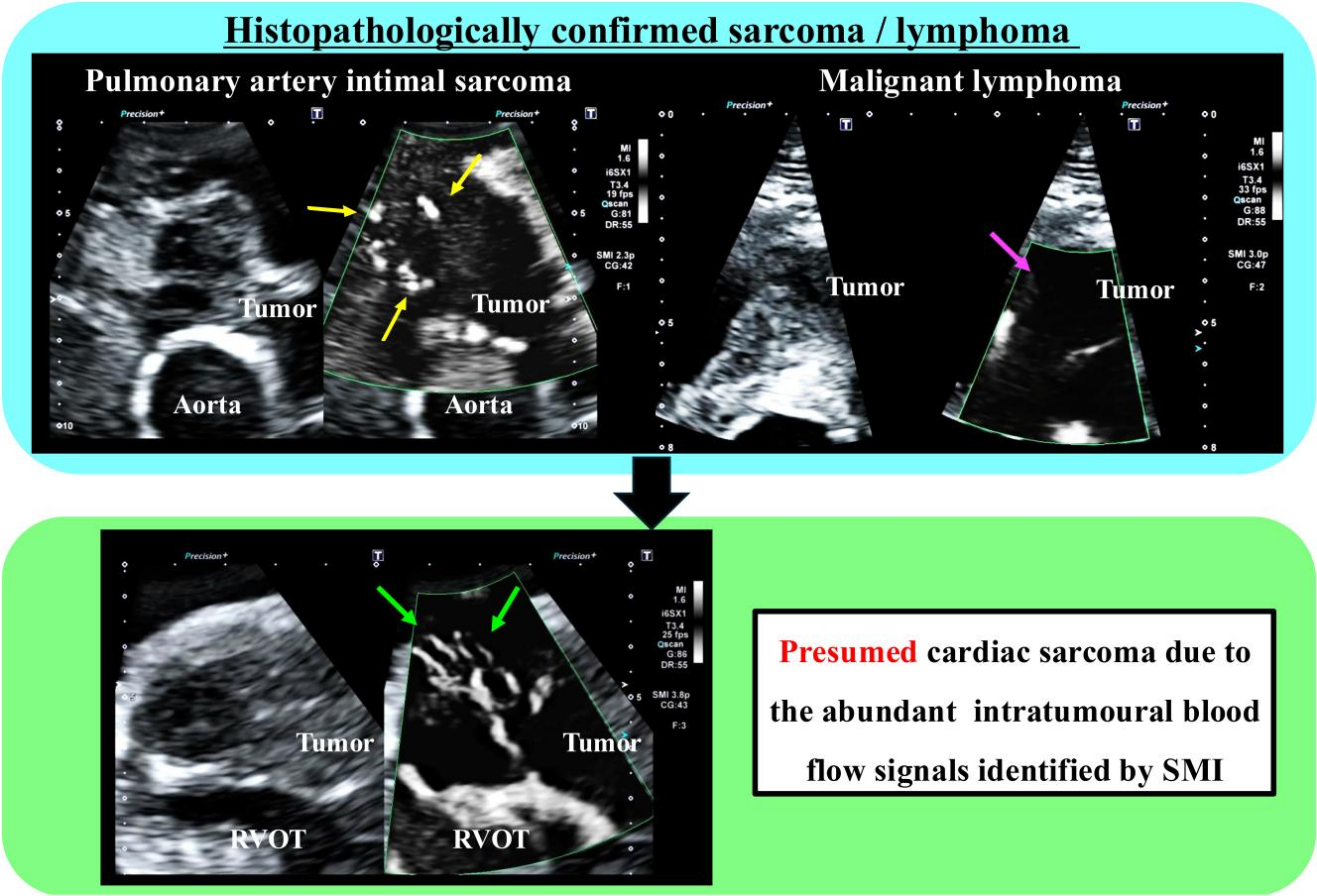


## Case 1

**Figure 1:**
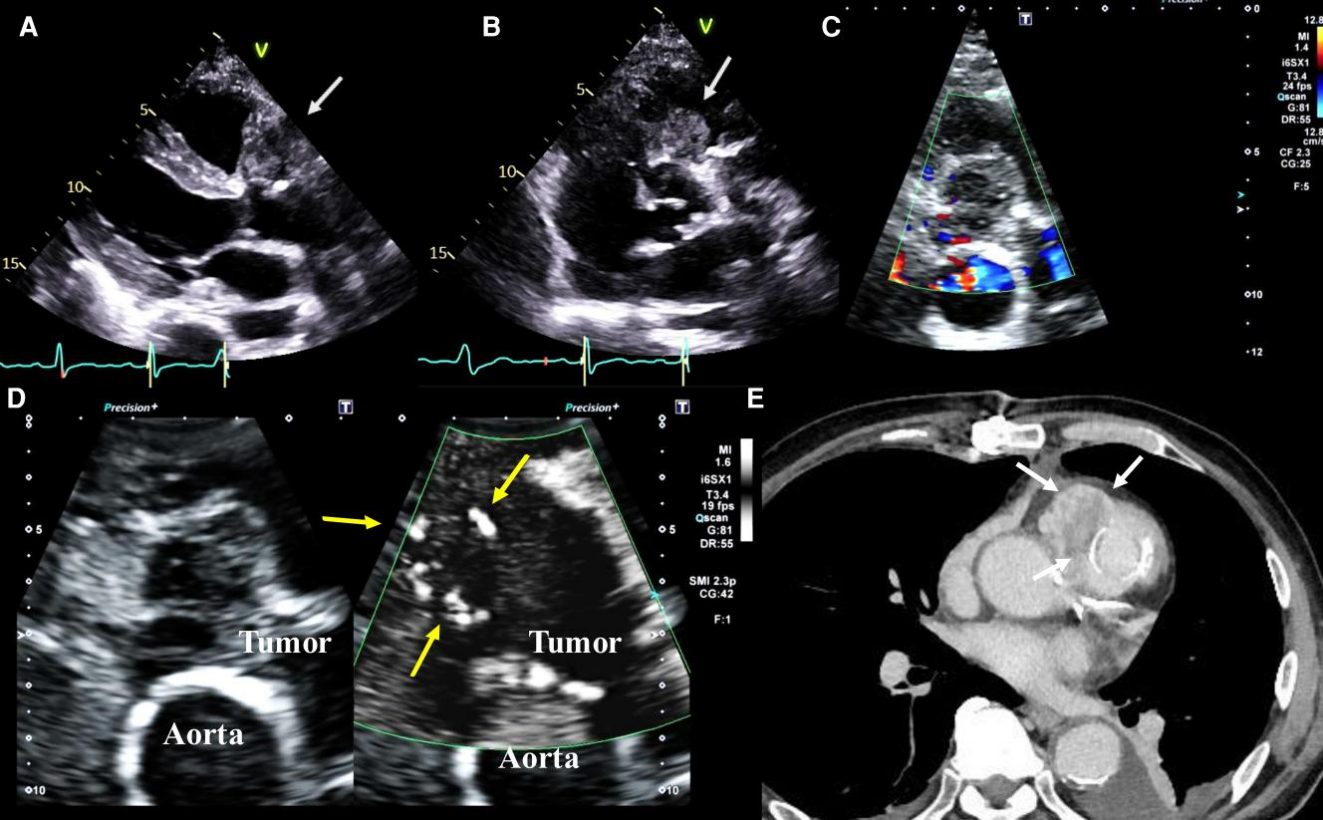
A case of pulmonary artery intimal sarcoma. Transthoracic echocardiography scans in a parasternal view (*A*) and a short-axis view (*B*) show a residual tumour measuring 31 × 32 mm in the right ventricular outflow tract (white arrows). (*C*) The intratumoural blood flow signal could not be clearly detected by conventional colour Doppler, even with a reduced velocity scale. (*D*) Superb microvascular imaging detected abundant intratumoural blood flow signals (yellow arrows). (*E*) Cardiac contrast-enhanced computed tomography scan showing a residual tumour with an irregular margin and a surrounding contrast effect in the right ventricular outflow tract (white arrows).

A 79-year-old man was referred to our hospital for further examination of a cardiac tumour. Transthoracic echocardiography revealed a large tumour with an irregular margin in the right ventricular outflow tract and pulmonary artery. The right ventricular outflow tract was severely obstructed by the large tumour. These findings of an irregular margin and invasion into surrounding tissues suggested that the tumour was a sarcoma, as the prevalence of sarcoma is high in malignant tumours of the heart. Tumour resection, pulmonary valve replacement, and patch augmentation of the pulmonary artery were performed to confirm the diagnosis and prevent occlusion of the outflow tract. The tumour was histologically diagnosed as a pulmonary artery intimal sarcoma. Postoperative transthoracic echocardiography using an i6SX1 probe (Aplio i900; Canon Medical Systems, Odawara, Japan) revealed a residual tumour measuring 31 × 32 mm in the right ventricular outflow tract (*Figure 1A* and *B*). The intratumoural blood flow signal could not be displayed clearly using conventional colour Doppler, even after reducing the velocity scale (*Figure 1C*). However, SMI could detect abundant intratumoural blood flow signals (scale: 10.4, gain: 42, frame rate: 19 fps, frequency: 2.3 MHz, filter: 1), which were compatible with residual sarcoma (*Figure 1D*) (see Supplementary material online, *Video S1*). Contrast-enhanced cardiac computed tomography (CT) subsequently demonstrated a residual tumour with an irregular margin and contrast enhancement of the right ventricular outflow tract, consistent with a diagnosis of sarcoma (*Figure 1E*). He was transferred to a university hospital and planned to administer palliative chemotherapy.

## Case 2

**Figure 2:**
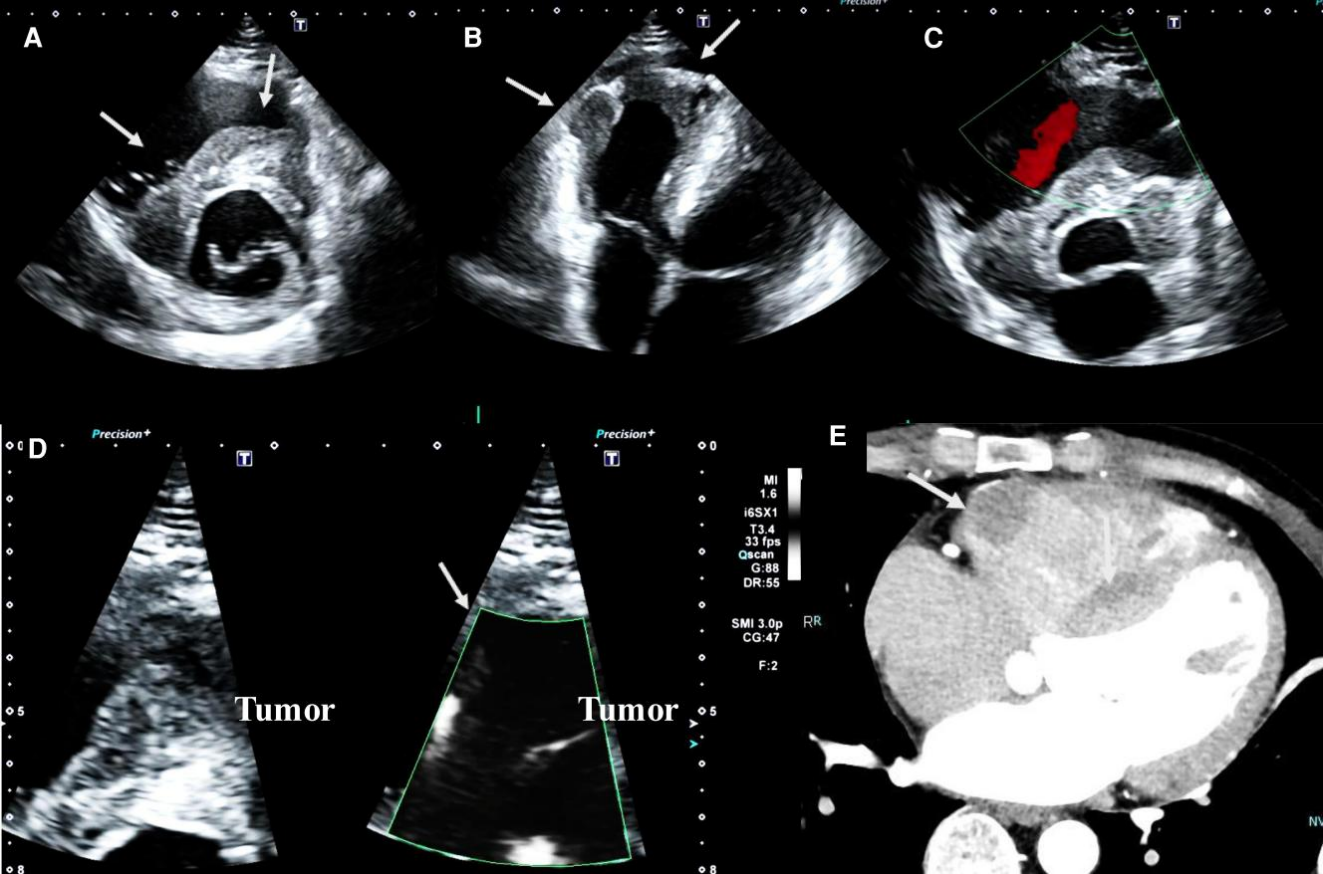
A case of malignant lymphoma. Transthoracic echocardiography scans in (*A*) a short-axis view and (*B*) a modified apical four-chamber view of the right ventricle showing intracardiac tumours with irregular margins in the right ventricular outflow tract and free wall of the right ventricle (white arrows). (*C*) Conventional colour Doppler and (*D*) superb microvascular imaging failed to detect any intratumoural blood flow signals (white arrows). (*E*) Contrast-enhanced cardiac computed tomography scan revealed lower contrast effects in the tumour (white arrows).

A 48-year-old man complained of dizziness, syncope, and intermittent chest pain and was referred to our hospital, where he was diagnosed with complete atrioventricular block and cardiac hypertrophy. Transthoracic echocardiography showed intracardiac tumours with an irregular margin in the right ventricular outflow tract and free wall of the right ventricle (*Figure 2A–C*). Intratumoural blood flow was evaluated using SMI, but no flow signals were detected (scale: 11.1, gain: 47, frame rate: 33 fps, frequency: 3.0 MHz, filter: 2) (*Figure 2D*) (see Supplementary material online, *Video S2*). Cardiac contrast-enhanced CT revealed that contrast enhancement was lower in the tumour than in the myocardium (*Figure 2E*), consistent with the SMI findings, which showed no intratumoural blood flow. Malignant lymphoma was suspected because of poor intratumoural blood flow. The tumour was histologically diagnosed as malignant lymphoma by endomyocardial biopsy of the right ventricle. He was transferred to the haematology department and underwent chemotherapy for malignant lymphoma.

## Case 3

**Figure 3:**
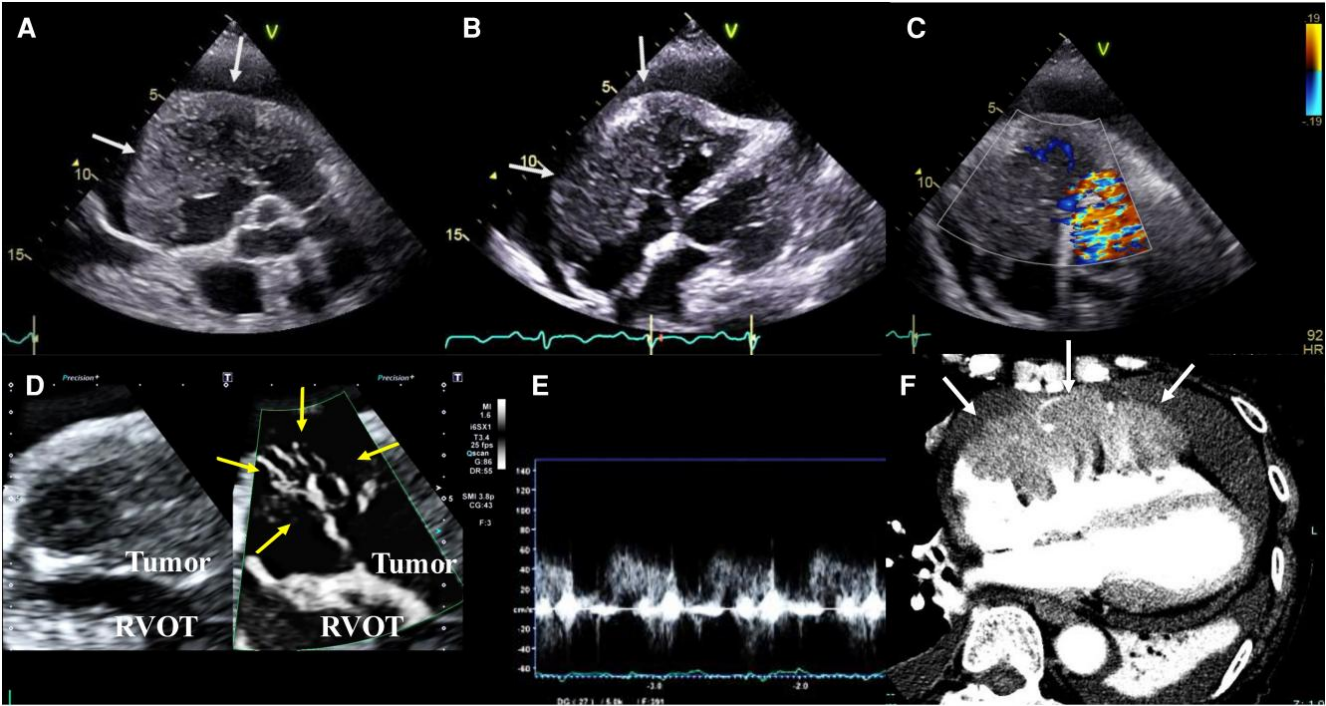
A presumed case of cardiac sarcoma. Transthoracic echocardiography scans in (*A*) a short-axis view and (*B*) a modified apical four-chamber view of the right ventricle showing intracardiac tumours with irregular margins in the right ventricular outflow tract, the free wall of the right ventricle, and the epicardium (white arrows). (*C*) Conventional colour Doppler with reduced velocity scale could not adequately display the intratumoural blood flow signal. (*D*) Superb microvascular imaging detected abundant intratumoural blood flow signals (yellow arrows). (*E*) Flow pattern in the tumour depicted using pulse Doppler: the blood flow signals are predominantly seen in diastole, similar to the pattern seen in a coronary artery. (*F*) Contrast-enhanced cardiac computed tomography scan showing contrast effects in the tumour (white arrows).

A 91-year-old woman with exertional dyspnoea was referred to our hospital for evaluation of a large tumour in the right atrium and ventricle. Transthoracic echocardiography revealed a low-isoechoic tumour with an irregular margin invading the epicardium and a large amount of pericardial effusion (*Figure 3A* and *B*). Even with a reduced velocity scale, conventional colour Doppler could not show the intratumoural blood flow signals (*Figure 3C*), but SMI could detect abundant intratumoural blood flow signals (scale: 11.1, gain: 43, frame rate: 25 fps, frequency: 3.8 MHz, filter: 3) (*Figure 3D*) (see Supplementary material online, *Video S3*). The intratumoural blood flow signals were predominantly shown in diastole, similar to those in a coronary artery (*Figure 3E*). It was thought that blood flow nourished the tumour. Cardiac contrast-enhanced CT also showed contrast enhancement in the tumour, compatible with the findings on SMI (*Figure 3F*). Therefore, sarcoma was suspected based on findings on transthoracic echocardiography and contrast CT. However, the patient was too old and frail to undergo surgery and biopsy or receive a full course of chemotherapy for sarcoma or malignant lymphoma, as well as even palliative chemotherapy for malignant lymphoma. Therefore, the best supportive care was selected based on the findings of SMI, without performing invasive tests or treatments.

The imaging findings across modalities (SMI, CT, and biopsy) for the three cases were summarized in *Table 1*.

**Table 1:** Imaging findings across modalities

	SMI	Contrast-enhanced CT	Biopsy (histological findings)
Case 1	Abundant intratumoural blood flow signals	Contrast effects in the tumour	Pulmonary artery intimal sarcoma
Case 2	Failing to detect any intratumoural blood flow signals	Lower contrast effects in the tumour compared with the myocardium	Malignant lymphoma
Case 3	Abundant intratumoural blood flow signals	Contrast effects in the tumour	Not performed

CT, computed tomography; SMI, superb microvascular imaging.

## Discussion

Most primary malignant cardiac tumours (more than 95%) are sarcomas, and the remainder are often malignant lymphomas or mesotheliomas.^4^ Cardiac sarcoma progresses rapidly and has a poor prognosis. The median survival time for patients with cardiac sarcoma is approximately 1 year, even with multidisciplinary treatment including surgery, chemotherapy, and radiotherapy.^5^ However, patients with cardiac malignant lymphoma who respond to chemotherapy have the possibility of a cure. Therefore, rapid differential diagnosis of these two cardiac tumours is essential when deciding on treatment, including best supportive care for cardiac sarcoma. A cardiac tumour is essentially diagnosed histologically by surgical biopsy. However, surgical biopsy is invasive, and non-invasive diagnostic accesses using echocardiography is clinically important for maintaining quality of life, especially in patients with cardiac sarcoma who have a very poor prognosis. In general, intratumoural blood flow is abundant in patients with cardiac sarcoma and poor in those with malignant lymphoma.^4,6^ In Case 3, invasive biopsy was avoided by identifying abundant intratumoural blood flow using SMI. This strategy may be beneficial for elderly patients who cannot undergo chemotherapy because of poor performance status.

There have been no reports on the usefulness of SMI for evaluating intratumoural blood flow in the heart, although its usefulness in patients with superficial or abdominal tumours has been reported.^7^ Unlike other organs, the heart repeatedly contracts and expands during the cardiac cycle, resulting in more motion artefacts. Therefore, it is difficult to evaluate low-velocity blood flow in cardiac tumours using conventional colour Doppler imaging. Our experience has been that SMI can assess the amount of intratumoural blood flow in a tumour and assist hypothesis generation in the diagnosis of cardiac sarcoma and malignant lymphoma by providing supportive haemodynamic characterization, although it should be acknowledged the following limitations: (i) lymphoma can be hypervascular exceptionally, (ii) sarcoma may contain necrosis with low flow, and (iii) thrombus can mimic avascular tumour. We had no experience assessing intratumoural blood flow in cardiac metastases, which are the most common cardiac tumours in elderly patients and frequently show heterogeneous perfusion. Considering the cases of superficial or abdominal tumours,^7^ intratumoural blood flow signals may be detected using SMI in cardiac metastases, allowing differentiation from malignant lymphoma; however, distinguishing between sarcoma and cardiac metastases may be difficult. Moreover, this report consists of a small number of cases and lacks a comparator imaging analysis of the tumour. Hence, it should be noted that SMI is not a definitive diagnostic modality for the differential diagnosis of tumours, but rather a minimally invasive modality that complements contrast echocardiography, CT, or cardiac magnetic resonance, which is semi-invasive and not available to all patients in an outpatient clinic. Evaluation of intratumoural blood flow using SMI provides supportive haemodynamic characterization for the differential diagnosis of cardiac tumours and may shorten the time to treatment.

## Supplementary Material

ytag343_Supplementary_Data

## Data Availability

The data underlying this article will be shared on reasonable request to the corresponding author.

## Lead author biography

Ayaka Mizumoto, MS, was born in Ehime, Japan, in 1988 and is a medical sonographer in the Japan Society of Ultrasonic Medicine. She is currently working in the Department of Biomedical Laboratory Scientist at National Cerebral and Cardiovascular Center, Osaka, Japan. Her clinical research interests include new technologies in adult cardiac echocardiography, such as 3D imaging and superb microvascular imaging (SMI), as well as exercise stress echocardiography.

## Notes

**Consent:** The authors confirm that written consent for submission and publication of this case report, including images, was obtained in accordance with COPE guidelines.
