# Supplementary figures and images for: Usefulness of superb microvascular imaging for differential diagnosis of malignant cardiac tumours: a case series
Source: Eur Heart J Case Rep. 2026 May 11;10(5):ytag343. doi: 10.1093/ehjcr/ytag343 (PMC13198372; doi:10.1093/ehjcr/ytag343)

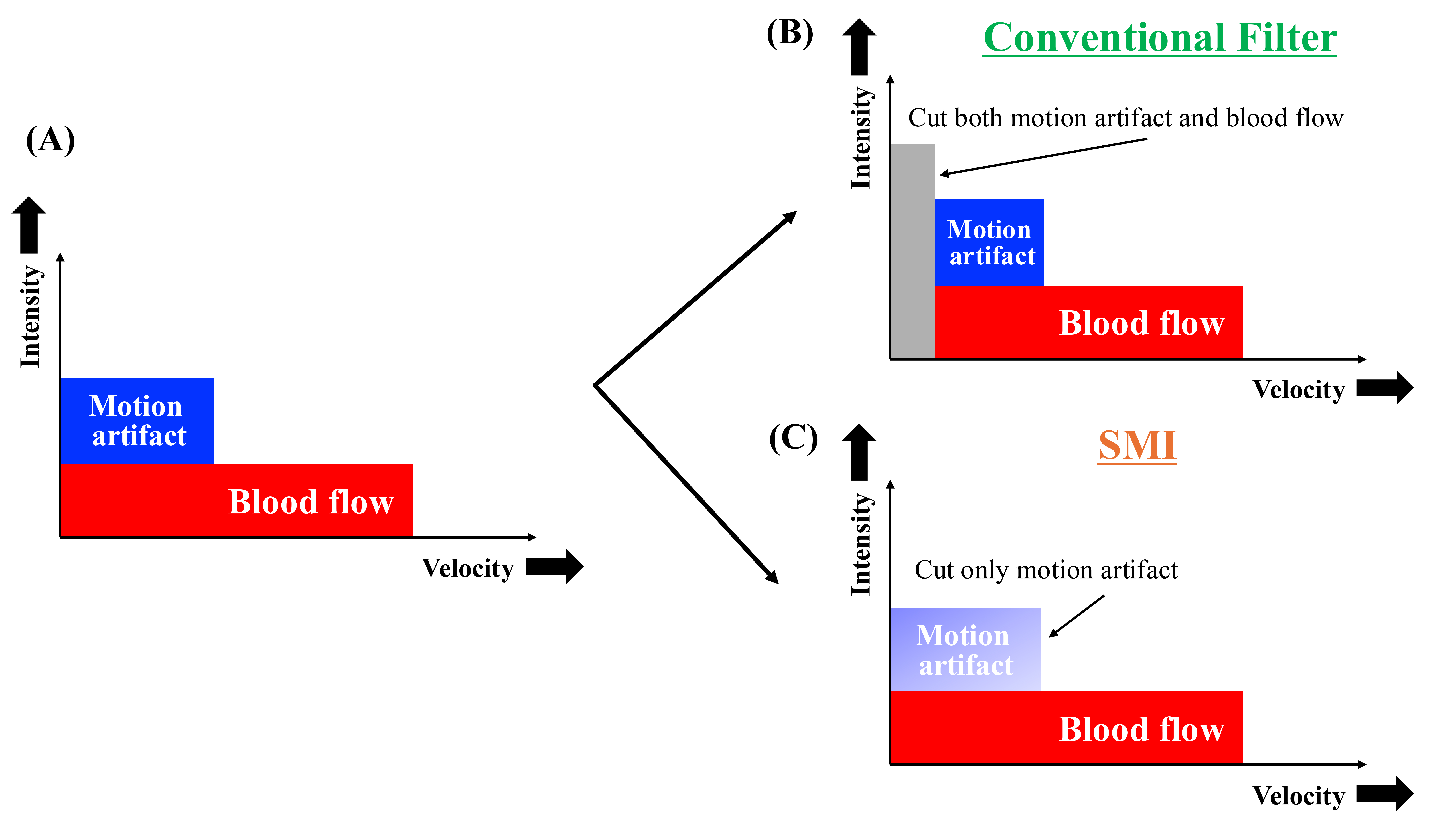

Supplement: ytag343_Supplementary_Data [file ytag343_supplementary_data.zip › Supplementary Figure 1.jpg]
